# Effect of Bee Venom and Its Fractions on the Release of Pro-Inflammatory Cytokines in PMA-Differentiated U937 Cells Co-Stimulated with LPS

**DOI:** 10.3390/vaccines4020011

**Published:** 2016-04-19

**Authors:** Jonans Tusiimire, Jennifer Wallace, Nicola Woods, Mark J. Dufton, John A. Parkinson, Grainne Abbott, Carol J. Clements, Louise Young, Jin Kyu Park, Jong Woon Jeon, Valerie A. Ferro, David G. Watson

**Affiliations:** 1 Strathclyde Institute of Pharmacy and Biomedical Sciences, University of Strathclyde, 161 Cathedral Street, Glasgow G4 0RE, U.K.; jonans.tusiimire@strath.ac.uk (J.T.); nicola.woods@strath.ac.uk (N.W.); grainne.abbott@strath.ac.uk (G.A.); c.j.clements@strath.ac.uk (C.J.C.); Louise.c.young@strath.ac.uk (L.Y.); v.a.ferro@strath.ac.uk (V.A.F.); 2 WestCHEM Department of Pure and Applied Chemistry, University of Strathclyde, 295 Cathedral Street, Glasgow G1 1XL, U.K.; jennifer.wallace.101@strath.ac.uk (J.W.); mark.dufton@strath.ac.uk (M.J.D.); john.parkinson@strath.ac.uk (J.A.P.); 3 #204, Beesen Co. Ltd., Bio Venture Town, Yuseong Daero 1662, 34054 Dae Jeon, Korea; jkypark@live.co.kr (J.K.P.); confessor@hanmail.net (J.W.J.)

**Keywords:** bee venom, pro-inflammatory cytokines, LPS stimulation, U937 cells, PMA differentiated, ELISA, (*Z*)-9-eicosen-1-ol, *Apis mellifera*

## Abstract

The venom of *Apis mellifera* (honey bee) has been reported to play a role in immunotherapy, but existing evidence to support its immuno-modulatory claims is insufficient. Four fractions from whole bee venom (BV) were separated using medium pressure liquid chromatography. Their ability to induce the production of cytokines TNFα, IL-1β and IL-6 in phorbol-12-myristate-13-acetate (PMA)-treated U937 cells was assessed. The levels of the three cytokines produced by stimulation with the four fractions and crude BV without LPS were not significantly different from negative control values. However, co-stimulation of the cells with LPS and Fraction 4 (F-4) induced a 1.6-fold increase in TNF-α level (*p* < 0.05) compared to LPS alone. Likewise, LPS-induced IL-1β production was significantly synergised in the presence of F-1 (nine-fold), F-2 (six-fold), F-3 (four-fold) and F-4 (two-fold) fractions, but was only slightly enhanced with crude BV (1.5-fold) relative to LPS. Furthermore, the LPS-stimulated production of IL-6 was not significantly increased in cells co-treated with F-2 and F-3, but the organic fraction (F-4) showed an inhibitory effect (*p* < 0.05) on IL-6 production. The latter was elucidated by NMR spectroscopy and found to contain(*Z*)-9-eicosen-1-ol. The effects observed with the purified BV fractions were more marked than those obtained with the crude sample.

## 1. Introduction

Bee venom (BV) is mainly used as a defence tool by the honey bee, and its primary function is to inflict pain on any intruders into the hive [1]. Despite its pain-causing effects, the main reported human administration uses relate to pain relief in conditions, such as arthritis and rheumatism [2,3], tendonitis, multiple sclerosis, wounds and gout [4,5,6]. The chemical composition of BV is complex, but the primary ingredients are bioactive peptides, proteins and several other biomolecules [6,7,8,9,10]. The principal component is a 26-amino acid haemolytic peptide, melittin, which accounts for about 50%–60% of the venom by dry weight and is responsible for most of the observed effects [9,11,12].

BV components have been reported to possess various and, sometimes, conflicting immune-related effects. Available evidence suggests that apamin [13], histamine [14], mast cell degranulating (MCD) peptide [15,16] and phospholipase A_2_ (PLA_2_) [17] significantly increase the inflammatory response. The small neurotoxic peptide apamin (MW 2.0 kDa) is a Ca^2+^-activated K^+^-channel blocker, which has been reported to increase murine T-cell proliferation [13]. However, it has also been reported to inhibit histamine release in lung tissues, suggesting that it could decrease allergic airway inflammation through mast cell stabilisation [18]. Park *et al.* (2004) also demonstrated that histamine increased the production of IL-6 in nasal fibroblasts and induced nuclear factor kappa B (NF-κB) [14], a transcriptional factor for many pro-inflammatory genes [19]. On the other hand, MCD peptide was reported to inhibit histamine release from mast cells [15] by binding, in a dose-related manner, to mast cell receptors, thereby partially inhibiting IgE binding to these receptors [20]. Similarly, PLA_2_ was shown to activate T-cells via its action on phospho-diacylglycerides to form small neoantigenic factors *in vivo*, in a process dependent on antigen presentation by CD1a proteins [17].

Conversely, some components of BV have been reported to possess anti-inflammatory actions. For instance, the basic polypeptide adolapin (MW 11.5 kDa) was reported to possess anti-inflammatory and analgesic activities in carrageenan-, prostaglandin- and adjuvant-induced rat oedema and adjuvant polyarthritis [21]. These effects were attributed to the inhibition of prostaglandin synthesis, via cyclooxygenase inhibition, as well as through central mechanisms [21]. Adolapin was also shown to inhibit the activity of BV PLA_2_ and human lipoxygenase from platelets and possessed antipyretic effects [22].

Past reports on immuno-modulating effects of the main bee venom peptide melittin are rather contradictory. For instance, whereas Bramwell *et al.* (2003) reported dose-dependent mucosal adjuvant action of intranasal melittin co-administered with tetanus and diphtheria toxoids in mice [23], a number of other studies have reported its “neutralising” effects on LPS in murine macrophage cell lines [24,25]. The adjuvant effects of melittin were linked to its enhancement of vaccine absorption across the mucosal lining, which led to higher antibody (IgG) titres than those of either component alone [23]. On the other hand, its antagonistic effects on LPS were linked to inhibition of NF-κB binding to DNA [24] and phosphorylation of IκB kinase [25], respectively. In addition, treatment of LPS-stimulated BV2 immortalized murine microglial cells with BV or melittin, decreased the expression of pro-inflammatory cytokines (IL-1β, IL-6 and TNF-α) and inhibited inducible nitric oxide synthase (iNOS) production and nitric oxide (NO) expression, as was the expression of cyclooxygenase-2 (COX-2) and prostaglandin E_2_ (PGE_2_) production [26,27]. These anti-inflammatory effects were linked mainly to the leucine zipper sequence in melittin, which contains two leucine residues, since Leu-Ala substitution in this sequence progressively reduced this neutralising effect [27]. Jang *et al.* (2005) also reported anti-inflammatory effects of BV in RAW 264.7 macrophages that were attributed to a downregulation of iNOS, COX-2, NF-κB and mitogen-activated protein kinases (MAPKs) [28]. In addition, Park *et al.* (2004) also reported that BV and melittin decreased carrageenan-induced oedema and adjuvant-induced arthritis in rat models consistent with their inhibitory effects on LPS-induced expression of COX-2, cytosolic PLA_2_ and iNOS and on the generation of PGE_2_ and NO [24]. BV and melittin also prevented LPS-induced transcriptional and DNA binding activity of NF-κB via inhibition of IκB release [2].

Since upregulation of most pro-inflammatory genes (e.g., cytokines and chemokines) relies on activation of NF-κB [19], inactivation of the latter by BV or its components would be a key mechanism for exerting anti-inflammatory effects [29]. However, in a previous study, no significant inactivation of IL-1β-induced NF-κB was observed in fibroblast-like synoviocytes from rheumatoid arthritis patients, as well as in dermal fibroblasts and red blood cells from healthy volunteers, after treatment with BV and melittin [30]. In addition, there was no effect on the phosphorylation or degradation of IκB, and even at high concentrations, BV and melittin had no effect on NF-κB-p50-DNA interactions. Instead, significant increases in mRNA levels of several pro-inflammatory genes (including COX-2, IL-1β, TNF-α) and large quantities of oxygen radicals were observed following exposure to BV or melittin [30]. This suggested that melittin alone and BV as a whole are pro- rather than anti-inflammatory.

In the current study, the effects of BV and its fractions on the production of cytokines TNF-α, IL-1β and IL-6 were investigated in PMA-treated U937 cells. The latter belong to a monocytic differentiation lineage derived from malignant cells of a patient with generalised histiocytic lymphoma [31], and their differentiation with PMA, a potent tumour promoting agent [32], is known to impart functional properties typical of macrophages [32,33,34]. The presence of synergy between BV and LPS, a standard antigen, in inducing the production of the pro-inflammatory mediators would suggest the potential application of BV as a source of immuno-modulating agents for use as vaccine adjuvants.

## 2. Materials and Methods

### 2.1. Cell Culture

U937 cell cultures (obtained from ECACC, Porton Down, Salisbury, UK) were seeded at 3 × 10^5^ cells/mL in RPMI-1640 (Lonza, Verviers, Belgium) supplemented with 2 mM l-glutamine (Life Tech, Paisley, UK), 100 IU/100 µg/mL penicillin/streptomycin (Life Tech, Paisley, UK) and 10% (*v*/*v*) foetal bovine serum (FBS) (Sigma-Aldrich, Dorset, UK). Cells were subcultured every 2–4 days and maintained at 37 °C in a humidified atmosphere of 5% CO_2_.

### 2.2. Test Sample Isolation, Preparation and Analysis

Crude BV (supplied lyophilized by Beesen Co. Ltd., Dae Jeon, Korea) was prepared for bioassay by dissolving 10 mg in 1 mL of dimethyl sulphoxide (DMSO, Sigma-Aldrich) followed by filtration through a 0.2-μm filter (Millex^®^, Sigma-Aldrich). The venom fractions F-1–F-4 were isolated from 800 mg of crude BV by reversed phase medium pressure liquid chromatography (MPLC) on a Reveleris^®^ iES flash chromatography system (Grace Davison Discovery Sciences, Carnforth, UK) with dual UV (λ = 220/280 nm) and evaporative light scattering (ELSD) detection. The sample was mixed with 3 g of Celite^®^ before loading it into a dry-loading cartridge. The column used was an Easyvarioflash D24 cartridge (VWR International, Lutterworth, UK) packed with *ca.* 13 g of Polymeric Retain PEP for SPE (Thermo Scientific, Paisley, U.K.), as previously described [35]. Fraction F-1 was eluted with 100% water and F-4 with 100% acetonitrile (Sigma-Aldrich), both solvents being of HPLC grade. Fractions F-2 and F-3 were eluted in water/acetonitrile mixtures of 80/20% and 50/50%, respectively. The resulting purified fractions were freeze-dried and stored at −30 °C until required for the assay. Samples for bioassays were reconstituted at 10 mg/mL in DMSO. Liquid chromatography-mass spectrometry (LC-MS) analysis of the freeze-dried fractions was carried out. The samples were reconstituted in water (F-1, F-2 and F-3) or acetonitrile:water (1:1) (F-4) to achieve concentrations of 0.1 mg/mL in each case, and 10-µL aliquots were injected into a Finnigan Surveyor HPLC system interfaced to an Orbitrap Mass Spectrometer (Thermo Fisher Scientific, Bremen, Germany). All of the samples were analysed using an ACE 3 C18 column (150 × 3.0 mm, 3 µm particle size) supplied by Hichrom Ltd. (Reading, UK). The mobile phase consisted of 0.1% formic acid in water (A) and 0.1% formic acid in acetonitrile (B) at a flow rate of 0.3 mL/min. The gradient used for F-1 to F-3 was 20%–70% B from 0–10 min, 6 min hold at 70% B, then return to 20% B over 4 min, followed by 5 min re-equilibration. In the case of F-4, an initial 5-min isocratic profile at 50% B was followed by a 1-min ramp to 95% B, held there for 8 min, before a 1-min return to 50% B and re-equilibration for 4 min. Full scan spectra were obtained within *m*/*z* 100–2000 in the positive ESI mode for all samples, except F-4, which was detected in the *m*/*z* range 200–1200 in the negative ESI mode. For MS/MS of F-4, collision-induced dissociation (CID) of the [M − H]^−^ parental ion was carried out at a normalised collisional energy of 35.0 V, and the product ion scan was made in the *m*/*z* rage of 200–700, also in the negative ESI mode. The spray needle voltages were set at 4.5 and −3.5 kV in positive and negative ESI modes, respectively. The sheath and auxiliary gas flow rates were 50 and 15 arbitrary units, respectively, while ion transfer capillary temperature was set at 275 °C. All data were collected and processed using XCalibur software (version 2.1.0, Thermo Fisher Scientific, Bremen, Germany).

### 2.3. Cytotoxicity Assay

U937 cells were seeded at 2.25 × 10^4^ cells/well in 96-well plates (Corning^®^, Sigma-Aldrich) and incubated in the presence and absence of BV or its fractions at final concentrations ranging from 100 µg/mL–3 ng/mL (*n* = 3). Triton X at 1% (*v*/*v*) served as a positive control. The plate was then incubated at 37 °C and 5% CO_2_ in a humidified atmosphere for 48 h. After incubation, Alamar^®^ Blue (AbD Serotec^®^, Kidlington, U.K.) was added at a final concentration of 10% in a total assay volume of 100 μL per well and the plate incubated for a further 6 h. Fluorescence readings of the plate were taken using a Perkin Elmer Wallac Victor^2^ 1420 Multilabel Counter (λ_Ex/EM_: 560/590 nm). All readings were corrected for background by subtracting the mean fluorescence of the Triton X wells. Cell viability was then calculated for each well as a percentage of fluorescence readings in the presence of test sample relative to the mean value of the negative controls. The resulting data were analysed with GraphPad Prism for Windows (version 4.03, GraphPad Software, San Diego, CA, USA, www.graphpad.com) to obtain dose-response curves for each sample and their corresponding mean inhibitory concentration (IC_50_) values.

### 2.4. Induction of Cell Differentiation

U937 cells were seeded at 4.5 × 10^4^ cells/well in a volume of 450 µL in 24-well tissue culture plates (Corning^®^, Sigma-Aldrich) (*n* = 3) in media containing 60 ng/mL PMA (Sigma-Aldrich). A control well containing cells in media without PMA was also included. The cells were then incubated in a humidified atmosphere at 37 °C and 5% CO_2_ for 48 h. Micrographs of the cells were taken after 24 and 48 h for evidence of differentiation.

### 2.5. Stimulation of Cytokine Release

After 48 h of differentiation, the media were aspirated and replaced with fresh media, without PMA, and the cells incubated for a further 24 h. At this point, samples of BV or fractions, with or without *Escherichia coli* (*E. coli*) LPS (Sigma-Aldrich), were then added from a separate sample dilution plate prepared using 10 mg/mL stock solutions (Table S1 in the Supplementary Materials). The final concentrations of the samples on the cell culture plate were 100 µg/mL (F-1 and F-2), 12 µg/mL (F-3 and BV) and 120 µg/mL (F-4), respectively. The final LPS concentration in the LPS-containing samples was 1 μg/mL.

### 2.6. Assessment of Cytokine Release

Three ELISA kits from R&D Systems (Abingdon, U.K.) were used to assess the release of interleukin (IL)-1β/IL-1F-2, IL-6 and tumour necrosis factor (TNF)-α from LPS-stimulated and non-stimulated U937 cells. The ELISA assay was carried out according to the kit manufacturer’s instructions, except that the colour substrate used (3,3’5,5’-tetramethylbenzidine, TMB) was from Sigma-Aldrich (Dorset, U.K.) and came as ready for use. The reaction was stopped with 2 N sulphuric acid (H_2_SO_4_) and the plate was read immediately at a 450-nm wavelength using a SpectraMax Pro 5 (Wokingham, U.K.) with wavelength correction by subtracting readings taken at 570 nm.

### 2.7. Data Analysis

Standard calibration curves were plotted by fitting the optical density data of TNF-α, IL-1β and IL-6 to 4-parameter logistic (4-PL) regression curves (Table S2 and Figures S1–S9 in the Supplementary Materials). Each of these standards was prepared in duplicate at each of the concentrations in the ranges recommended by the manufacturer. The 4-PL regression equation is given by:

(1) $$y=d+\frac{a-d}{1+{\left(\frac{x}{c}\right)}^{b}}$$

where *y* is the response value (*i.e.*, measured optical density), *x* is the concentration (in pg/mL) and *a*, *b*, *c* and *d* are constants. The regression analysis also computes the *R*^2^ value, which gives an indication of how best the fitted curve agrees with the data. From Equation (1), the unknown concentration, *x*, of a sample of optical density, *y*, can be calculated according to:

(2) $$x=c{\left(\frac{a-d}{y-d}-1\right)}^{\frac{1}{b}}$$

Using Equation (2), the concentrations of TNF-α, IL-1β and IL-6 induced by each of the samples assayed (with and without LPS) were calculated and expressed as ratios of the mean cytokine level induced by LPS (positive control), assayed in triplicate (*n* = 3). The resulting data were then analysed with GraphPad Prism to obtain bar graphs whose statistical significances were tested at the 95% confidence level (CI) relative to the mean positive control ratio of 1.0.

### 2.8. NMR Spectroscopy

NMR spectroscopy was carried out on 7.4 mg of BV fraction F-4 dissolved in 600 μL DMSO-*d*_6_. NMR data were acquired with a Bruker AVANCE II^+^ NMR spectrometer (Bruker Biospin GmbH, Rheinstetten, Germany) equipped with a 14.1 T Ultrashield^TM^ superconducting magnet operating at a ^1^H resonance frequency of 600.13 MHz under TopSpin (version 2.1, Bruker Biospin GmbH, Rheinstetten, Germany) running in a Microsoft Windows environment. All data were acquired using a BBO-z-atm probe head operating at ambient temperature (298 K) regulated by means of a BCU-05 chiller unit.

1D ^1^H-NMR spectra were acquired with 16 transients over a frequency width of 7.2 kHz (12.0 ppm) centred at a frequency offset of 5.0 ppm into 32 K data points for an acquisition time of 2.27 s using a 30-degree radio frequency (r.f.) pulse and a recycle delay of 2.0 s.

1D ^13^C-{^1^H} NMR spectra were acquired with 1024 transients over a frequency width of 33.33 kHz (220.8 ppm) centred at a frequency offset of 100.0 ppm into 32 K data points for an acquisition time of 491.5 ms using a 30-degree r.f. pulse with continuous composite pulse decoupling applied at the ^1^H resonance frequency and using a recycle delay of 0.7 s.

Complete details for all NMR experimental conditions can be found in the Supporting Information.

## 3. Results

### 3.1. Composition of Fractions 1–4 from MPLC

From LC-MS analysis, the fractions were revealed to contain mixed components in F-1 and F-2, while F-3 and F-4 both contained largely single components. The major constituents of F-1 were putatively identified to be histamine, proline, noradrenaline, 5-aminovaleric acid, cellobiose and arginine (Figure S10). Fraction F-2 contained mainly PLA_2_, as well as varying amounts of apamin, secapin and MCD peptide (Figure S11). On the other hand, melittin was the principal component of F-3 (96% purity) (Figure S12), while the organic fraction, F-4, mainly contained a new compound identified through NMR analysis as (*Z*)-9-eicosen-1-ol and trace levels of an unidentified phospholipid. The LC-MS did not detect (*Z*)-9-eicosen-1-ol due to its absolute lack of ionisation in both positive and negative ESI modes, but instead detected the trace phospholipid impurity, undetected by NMR at its concentration in the fraction (Figure S13).

### 3.2. Cytotoxicity of BV Fractions against U937 Cells

**Figure 1:**
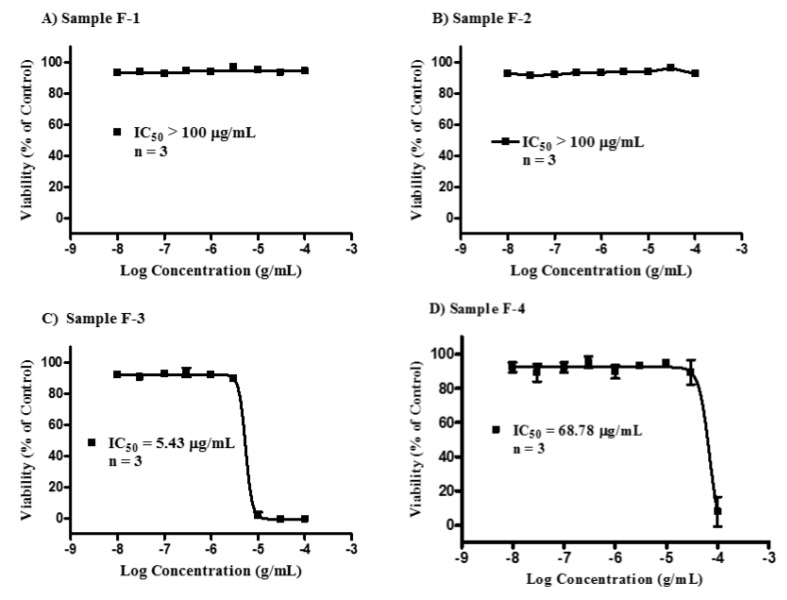
Cytotoxicity of the bee venom sample fractions F-1–F-4 against U937 cells. Fraction samples F-1 (**A**) and F-2 (**B**) were non-cytotoxic, each with an IC_50_ value >100 μg/mL. Fraction sample F-3 (**C**, melittin) was the most cytotoxic with an IC_50_ of 5.43 (95% CI 4.43–6.66) µg/mL, while fraction sample F-4 (**D**, lipid) had an IC_50_ value of 68.78 µg/mL.

**Figure 2:**
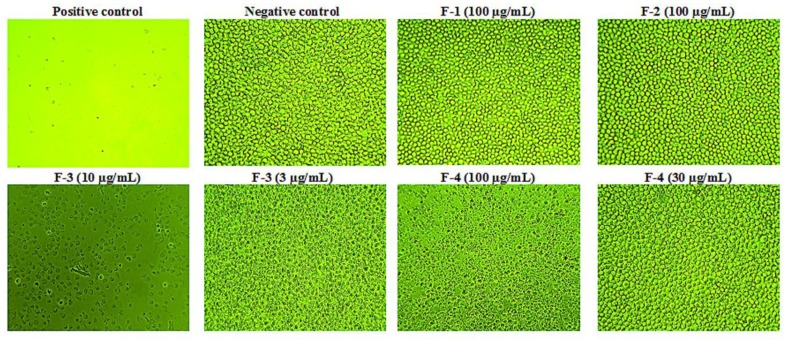
Cytotoxic effects of bee venom fractions on U937 cells. Fractions F-1 and F-2 were non-cytotoxic at the highest concentration of 100 µg/mL used, while F-3 was the most cytotoxic on the cells, as necrotic effects were observed even at 10 μg/mL (IC_50_ 5.4 µg/mL). On the other hand, F-4 was cytotoxic above 30 μg/mL with an IC_50_ of 68.8 μg/mL (*n* = 3). (Magnification = ×100).

Cytotoxicity studies were carried out (*n* = 3) to obtain IC_50_ values (Figure 1). Samples F-1 and F-2 gave IC_50_ of greater than 100 µg/mL. In contrast, F-3 gave the lowest IC_50_ value at 5.4 µg/mL (or 1.9 μM). The IC_50_ value of F-4 was 68.8 µg/mL. Micrographs of the cells taken after 24 and 48 h confirmed the assay results obtained with the Alamar^®^ Blue assay (Figure 2). These micrographs also revealed significant microscopic differences in the appearance of cells treated with F-3 and F-4 even in wells where Alamar^®^ Blue readings were comparable. Unlike the necrosis caused by melittin, which revealed the cells to have burst to release their protoplasm, non-viable F-4-treated cells appeared to have an intact cell outline, implying that the mechanism by which the lipid exerts its cytotoxic effect on U937 cells may be different from that of melittin, which acts through cell lysis [36].

### 3.3. Selection of BV and Fraction Concentrations for the Assay

ELISAs were carried out in order to determine the effect of the fractions on PMA-differentiated cells with respect to the production of three inflammatory mediators TNF-α, IL-1β and IL-6. Since the investigation of immuno-modulatory effects had to be conducted using concentrations of the fractions where the cells remained viable, concentrations were selected for each fraction that were below their respective IC_50_ values. Specifically, the highest final concentration of the fraction at which no toxicity was observed on the cells was used. Thus, F-1 and F-2 were each assayed at 100 μg/mL, F-3 and BV at 3 μg/mL, while F-4 was assayed at 30 μg/mL (Table S3 in the Supplementary Materials). The mean viability (*n* = 3) of the cells at the concentrations selected for each of the fractions used for the assay were F-1 (93%), F-2 (94%) and F-3 and F-4 (90% each), respectively, relative to the media control.

### 3.4. Effect of PMA on the U937 Cells

**Figure 3:**
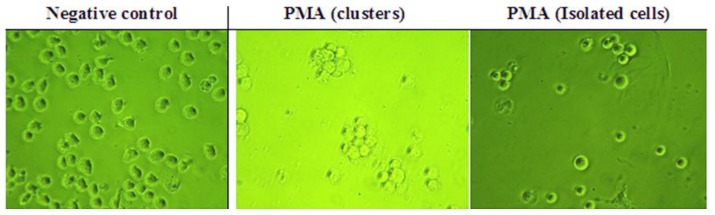
Effect of phorbol-12-myristate-13-acetate (PMA) on U937 cells. PMA was added to the cells at 60 ng/mL (*n* = 3). (Magnification = ×400).

After the cells had been incubated in the presence of PMA, they were observed microscopically at 24 and 48 h for the presence of features that confirmed whether or not they had differentiated [37]. Micrographs were also taken of the treated cells and compared to those of U937 cells in control wells (absence of PMA) on the same plate (Figure 3), which confirmed the morphological changes expected.

### 3.5. Effect on TNF-α Production

**Figure 4:**
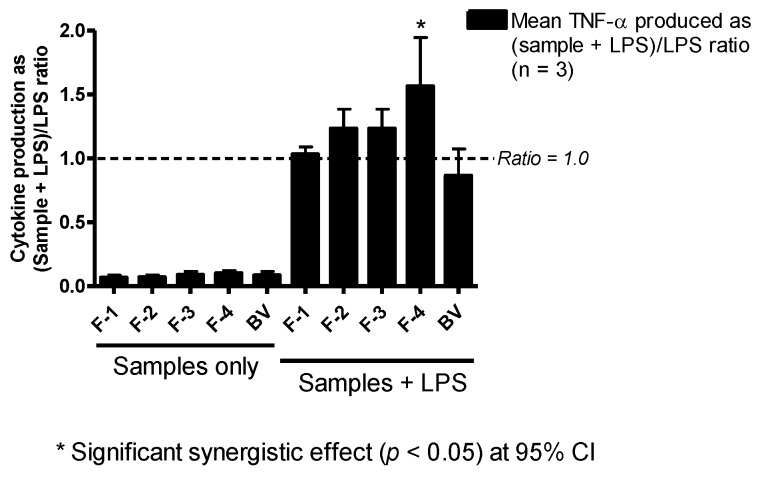
Effect of bee venom and its fractions of TNF-α production in PMA-differentiated U937 cells. All five samples tested produced slightly more than background levels of TNF-α, but not significantly different from those of the negative control (media). The level of TNF-α was significantly higher in fractions co-stimulated with F-4 and LPS compared to LPS, but the other fractions did not show any significant changes in the levels of the cytokine (*n* = 3).

The BV fractions on their own did not induce significant TNF-α production in PMA-differentiated U937 cells relative to the negative control (culture media). However, when used in combination with LPS, there was a noticeable enhancement (ratio >1.0) in the amount of TNF-α produced compared to LPS alone. This was statistically significant (*p* < 0.05) only with F-4, which produced a 1.6-fold increase in TNF-α release from the cells (Figure 4).

### 3.6. Effect on IL-1β Production

**Figure 5:**
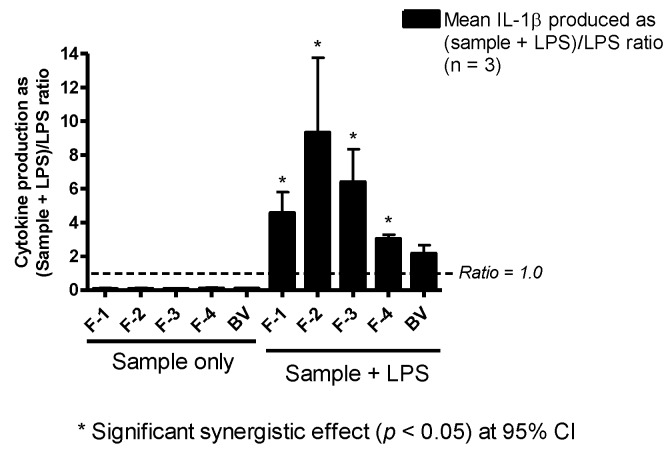
Effect of bee venom and its fractions of IL-1β/IL-1F2 production in PMA-differentiated U937 cells. All five been venom (BV) fractions tested produced only background levels of IL-1β/IL-1F2 when used alone. In the presence of LPS, there was significant synergy, especially with F-2 and F-3, which resulted in a nine- and six-fold increase in the production of the cytokine, respectively. Significant synergy was also observed with F-1 (four-fold) and F-4 (three-fold), but not with BV despite a two-fold increase in the latter (*n* = 3).

The enhancement of IL-1β/IL-1F2 by BV fractions in LPS co-stimulated U937 cells was much more pronounced than that observed with TNF-α. Fractions F-2 and F-3 greatly enhanced IL-1β/IL-1F2 release in the cells by approximately nine- and six-fold, respectively. Additionally, F-1 (four-fold), F-4 (three-fold) and whole BV (two-fold) also enhanced the release of this cytokine with LPS co-stimulation, although the increase obtained with BV was not statistically significant (Figure 5). As can be seen from Figure 5, the fractions on their own did not induce any significant level of cytokine release.

### 3.7. Effect on IL-6 Production

**Figure 6:**
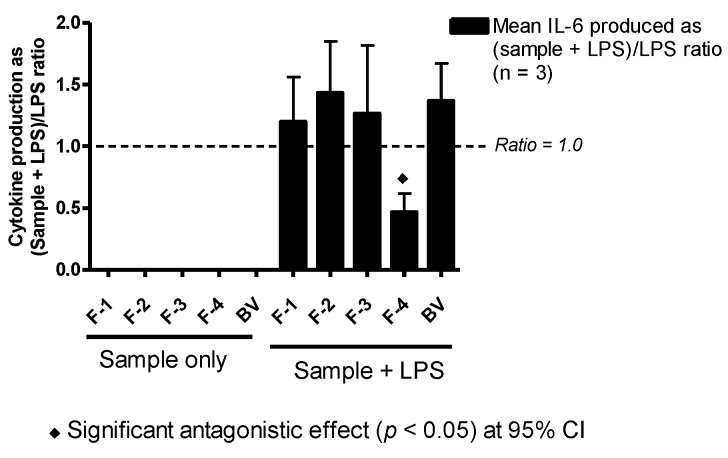
Effect of BV and its fractions with and without LPS on IL-6 production in PMA-differentiated U937 cells. The level of IL-6 produced by BV fractions alone was undetectable. However, in combination with LPS, Fractions F-1–F-3 and BV enhanced the level of cytokine produced by LPS though not significantly. Interestingly, F-4 significantly inhibited cytokine production by about 50% of the mean positive control (LPS) value (*n* = 3).

As with TNF-α and IL-1β production, the amount of IL-6 produced by the cells in the presence of BV fractions alone was undetectable (F-1–F-3) or not significantly different from levels observed in negative controls (F-4 and BV). Yet, when the same fractions were incubated together with LPS, the amount of IL-6 produced by the cells was raised, compared to that of stimulation with LPS alone, by 20%, 40%, 30% and 30% with F-1, F-2, F-3 and BV, respectively; although these were not significantly different from positive control values. Surprisingly, and contrary to observations with TNF-α and IL-1β, F-4 significantly decreased the amount of IL-6 released with LPS co-stimulation in the PMA-differentiated U937 cells by about 50% (Figure 6).

### 3.8. Identification of Active Compound in BV Fraction F-4

Given its unusual effect on TNF-α and IL-6 release in PMA-differentiated U937 cells, we sought to identify the component present in F-4 by NMR spectroscopy. The one-dimensional (1D) ^1^H-NMR spectrum of F-4 (acquired at 298 K in DMSO-*d_6_*; Figure S14) gave 10 distinguishable signals with chemical shifts and integrals as detailed in Table 1. Signal A, which corresponded to a ^1^H chemical shift associated with a proton attached to an sp^2^-hybridized carbon centre (alkene), integrated to two proton equivalents. Multiplicity-edited 2D [^1^H, ^13^C] HSQC-NMR data revealed that the signal was associated with a methine (CH) group, allowing the conclusion to be drawn that the molecule was likely to be a structure with close to two-fold symmetry about a double bond. The signal envelope designated H integrated to twenty two proton equivalents and suggested the presence of long chains of methylene groups typical of a lipid.

To establish how many types of carbon centres existed within the molecule, ^13^C-{^1^H} NMR data (Figure S15) were examined. The data gave rise to 16 NMR signals corresponding to sixteen different types of ^13^C environments. Many of these showed similar chemical shifts (signals e–l). Additionally the intensities revealed that a number of carbon centres (a, f, l, m) were twice as abundant, resulting from symmetry within the structure. It was clear in particular from these data that the carbon signal (a) was due to two alkene carbons with identical chemical shifts, confirming the expectation that the structure would be roughly symmetrical about a central double bond, the symmetry of which would remain largely unaffected by remote tail groups.

2D [^1^H, ^13^C] HSQC-NMR data (Figure S16) at both low and high resolution allowed the types of carbon to be distinguished for every centre, as well as editing the data to reveal protons that were not attached to carbon. As well as enabling the identification of H/C correlations within each magnetic environment, these data also made it possible to confirm the number of protons associated with the lipid chain. These data also revealed that protons giving rise to Resonances B and D were not attached to carbon. By analogy with literature examples, it was clear that Resonance D was associated with water in DMSO and could therefore be discounted from the analysis. A summary of the ^13^C-NMR data are shown in Table 2. These data are summarized to provide a molecular formula of C_20_H_40_O, yielding a molecular weight (MW) = 296. Integration of the ^1^H-NMR spectrum is consistent with this formula and the number of protons “counted” using the 2D [^1^H, ^13^C] HSQC-NMR data.

Analysis of the remaining 2D [^1^H, ^1^H] COSY and TOCSY and 2D [^1^H, ^13^C] HSQC and HMBC-NMR data (Figures S17–S19) is summarized in Table S7 and Table S8, respectively, in the Supplementary Materials. The triplet character of ^1^H Resonance B and its correlation with Resonance C by COSY and TOCSY indicated that the hydroxyl group was a terminal –OH attached to a methylene, whose protons gave rise to Resonance C. The triplet character of proton Signal I similarly enabled the identity of this resonance to be associated with a terminal methyl group. Correlations were traced as far as possible from both the terminal positions and the alkene proton resonances (A) until these assignment pathways merged at Resonance H. 2D [^1^H, ^13^C] HMBC-NMR data were used to establish longer range H/C correlations to reinforce the assignments, which remaining incomplete owing to the degeneracy at Signal H.

Following identification of coupling partners and piecing the structural evidence together, the proton and carbon assignments were allocated to a basic structure, as shown (Figure S20). It was not clear from the NMR data whether the double bond would be at the 9- or 10-position (shown in the 10-position in Figure S20). Neither was it clear from the data whether the double bond was of *E* or *Z* configuration. For this reason, simulations of the data were carried out based on both *E* and *Z* isomers of 9- and 10-eicosen-1-ol in order to throw some light on the conformation. Particular attention was paid to the appearance of proton Resonances A and E in these simulations, which would reflect directly on the conformation about the centralised double bond. The results of these simulations with their equivalent experimental counterparts are shown in Figure S21.

On balance, these data suggest a greater likelihood of the lipid existing in the (*Z*)-configuration, as shown in Figure S22. The position of the double bond is not revealed through these simulations or by experiment. Comparison with information provided directly through Beesen Co. Ltd., suppliers of the venom, suggests that the material and data are consistent with (*Z*)-eicos-9-en-1-ol.

### 3.9. Identification of Minor Component in F-4

Fraction F-4 was found to contain trace levels of an unidentified minor component with the [M − H]^−^ elemental composition of C_43_H_70_O_11_P (0.0783 ppm mass tolerance) and MW 794.47. Collisional induced dissociation (CID) of the parental ion (*m*/*z* 793.46) at a normalised collisional energy (NCE) of 35.0 produced two daughter ions, one with *m*/*z* 493.2574 (C_23_H_42_O_9_P, 2.685 ppm mass tolerance, 40%), possibly suggesting a loss of eicosapentenoic acid (MW 302), and the other with *m*/*z* 643.3608 (C_33_H_56_O_10_P, 0.341 ppm mass tolerance, 60%) (Figure S13). This elemental composition, though inconclusive, suggested the likelihood that the unknown impurity might be a phospholipid.

## 4. Discussion

### 4.1. Cytotoxicity

The two fractions that were cytotoxic to U937 cells with IC_50_ values below 100 µg/mL contained melittin (F-3, IC_50_ 5.4 µg/mL) and (*Z*)-9-eicosen-1-ol (F-4, IC_50_ 68.8 µg/mL). The latter also contained trace levels of an unidentified phospholipid. F-1 and F-2 were relatively non-toxic to U937 cells at the concentrations tested. The former contained mainly low MW amines, such as histamine, dopamine and noradrenaline, while the latter contained mainly PLA_2_, a major BV allergen [38,39]. Although one would have expected fraction F-2 to be cytotoxic due to its PLA_2_ content, it may be that the enzymatic activity reduced because of its separation from melittin (*i.e.*, the venom PLA_2_ and melittin act synergistically [40]) or as a result of loss of its 3D configuration during the fractionation. Fraction F-2 also contained variable amounts of the peptides apamin, MCD peptide and secapin, which were also detected in trace amounts in F-1.

Whereas the cytotoxicity of melittin is generally known in normal human and cancer cells [36,41], the biological activities of the organic fraction of BV are less well known. Melittin’s cytotoxicity is thought to be due to membrane-disruption [42] and apoptotic actions mediated via mitochondrial and caspase activities [43]. During our experiments, the toxicity of F-4 (at 100 μg/mL) was also observed in adherent normal human fibroblast (HS27) cells in which growth inhibition appeared to be associated with loss of cell attachment to the cell culture well plate (data not shown). Because of this, it had been anticipated that with suspended U937 cells, the F-4 fraction would have no such cytotoxic effect below 100 µg/mL, since adherence was not a prerequisite for cell division and growth. Thus, the observed toxicity in U937 cells might suggest that F-4 acts within the cell, at least partially, rather than exclusively externally to it. Its amphiphilic structure would be consistent with an ability to penetrate and pass through cell membranes.

### 4.2. Effect on Cytokine Release

The enhancement of LPS-stimulated release of IL-1β in U937 cells was by far the most pronounced effect induced by all four BV fractions and crude BV, while the effects on TNF-α and IL-6 release were less marked, with variability between the fractions. The only significant effect on TNF-α release was due to F-4, while F-2 and F-3, which contained PLA_2_ and melittin, respectively, were the most potent enhancers of IL-1β release by the cells following co-stimulation with LPS. Interestingly, F-4 showed anti-IL-6 effects. The IL-1 family of cytokines is closely linked to innate inflammatory and immune responses more than any other cytokine family, and IL-1β mediates auto-inflammatory diseases [44]. In the context of bees, the observed several-fold enhancement of IL-1β release by BV and all of its fractions is thus logical given the defensive function of the venom. Stimulation of IL-6 production is a key target for adjuvants due to its role in promoting B-lymphocyte differentiation into antibody-producing cells [45,46,47], T-cell proliferation [48] and development of cell-mediated cytotoxicity by CD8+ cells [49,50,51]. Thus, inhibition of IL-6 production by F-4 would suggest an immuno-suppressive action, but its concomitant stimulatory effect on TNF-α, an important cytokine involved in the development of resistance to infection and cancer with roles in necrosis and apoptosis [52], suggests a more subtle mechanism.

Among the major components of F-1, histamine was reported to increase production of IL-6, expression of histamine receptors, expression of the kinases pp38, pERK and pJNK and induction of NF-κB in nasal fibroblasts when assayed at 200 µM (~22.2 µg/mL) [14]. This concentration level was significantly higher than that present in the assay solutions of both the F-1 fraction and BV, which might explain their non-significant effects on IL-6. Bee venom PLA_2_, a major allergen and main component of F-2, and apamin have been shown to possess immune-inducing effects by activating T-cells [17] and promoting T-cell proliferation [13], respectively. Thus, the effects observed with F-2 on IL-1β and IL-6 production might be related to the effects of both PLA_2_ and apamin on the cells.

Additionally, melittin, the sole component of F-3, has been reported to possess adjuvant properties by enhancing the absorption of intranasal tetanus and diphtheria toxoids [23]. This would support its effect on IL-1β observed in this study. However, these findings do not suggest that melittin could reduce the effect of LPS on cells, contrary to some previous studies [26,27]. The concentration used in the current study (3 μg/mL) was sub-lethal to the U937 cells, but at 10 µg/mL, the concentration used in a previous study [27], melittin was found in the current study to be 100% cytotoxic. This was observed previously in dermal fibroblasts, mononuclear cells and fibroblast-like synoviocytes [30]. On the other hand, the study by Moon *et al.* [26] assayed both BV and melittin at 0.5–2.0 µg/mL, levels that are all markedly lower than those employed in the current study. In the same study, LPS was assayed at 0.5 µg/mL compared to 1.0 µg/mL used in this study, and the cells were initially treated with BV or melittin for 1 h before treatment with LPS, rather than being simultaneously exposed [26]. Thus, the differences observed in these *in vitro* studies in relation to melittin’s role in immuno-modulation might be related to the different experimental designs and/or concentrations of melittin and LPS used. In a previous study by Stuhlmeier (2007), neither BV nor melittin was found to inhibit IL-1β-induced activation of NF-κB. Instead, there were significant increases in the levels of the mRNA of several pro-inflammatory genes and COX-2 in synoviocytes, dermal fibroblasts and mononuclear cells [30]. The results obtained in our study corroborate these findings.

The main component of F-4, identified in this study as (Z)-9-eicosen-1-ol, resembled the lipid-soluble compound reported by Pickett *et al.* (1982) in *A. mellifera* venom and structurally elucidated as (Z)-11-eicosen-1-ol [53]. The latter was described as a natural pheromone, which acted synergistically with amyl acetate, another pheromone produced by the bees [53]. Schmidt *et al.* (1997) also reported the same long chain monounsaturated alcohol to be the main component of the oily fraction of *Apis cerana* venom [54], a species related to *A. mellifera*. The compound isolated in F-4 differs from that previously described with respect to the double bond position, which might be a means of conveying subtle differences in message recognition among the bees [55].

## 5. Conclusions

Inflammatory responses mediated by pro-inflammatory cytokines are a key component of protective immunity against many infections. This study shows that treatment of PMA-differentiated U937 cells with BV fractions significantly enhances the IL-1β cytokine release effect of LPS in these cells. However, neither BV nor its fractions could induce any significant cytokine release on their own. The largest synergistic effect was observed for IL-1β release, which was promoted by all fractions, while only the lipid fraction, F-4, enhanced TNF-α production in the cells co-stimulated with LPS. Although fraction F-4, identified to contain (*Z*)-9-eicosen-1-ol, was stimulatory for IL-1β and TNF-α release, it produced an inhibitory effect on IL-6 production. The commercial availability of this compound in larger amounts than can be isolated from the venom will allow us in future work to explore potential synergies between it and fractions F-1–F-3. In addition, it provides a lead compound for exploring the effects of other long chain alcohols, since they are accessible via reduction of the wide range of long chain fatty acids, that is commercially available. Taken together, these results do not support some studies in the literature that suggest that BV and melittin possess potential anti-inflammatory activity by antagonising LPS-stimulation of cytokine production. Instead, BV fractions synergise with LPS in the induction of the IL-1β cytokine release in U937 cells, and the lipophilic fraction has additional orthogonal effects on TNF-α and IL-6, whereby it induces the former and inhibits the latter. Overall, these effects provide valuable preliminary information to support further evaluation of purified BV as a potential source of natural adjuvants for some vaccines.

## Figures and Tables

**Table 1:** Data arising from the 1D ^1^H-NMR spectrum of the lipid component. Reference to the “link to ^13^C” arises from analysis of the 2D [^1^H, ^13^C] HSQC-NMR spectrum that reveals ^1^*J*_HC_ correlations where labels a–q correspond to the ^13^C resonances shown in (Figure S12).

Label	δ (ppm)	Integral	Type	Multiplicity	Link to ^13^C	Proposal
A	5.32	2	Alkene CH	Second order	a	Symmetric Alkene
B	4.30	1	-OH	t	-	-CH_2_-OH
C	3.36	2	-CH_2_-	dt	b	
D	3.32	1.7	H_2_O	s	-	Water in DMSO
E	1.98	4	-CH_2_-	q	m	
F	1.39	2	-CH_2_-	pentet	c	
G	1.29	4	-CH_2_-	q	f	
H	1.24	22	-CH_2_-	-	d,e,g,h,i,j,k,l,n,o	
I	0.85	3	-CH_3_	t	p	-CH_2_-CH_3_

**Table 2:** Summary of ^13^C-NMR data for the lipid molecule as revealed by 1D ^13^C-{^1^H} and 2D [^1^H, ^13^C] HSQC-NMR data.

Label	δ (ppm)	No. of Carbons	Type	Link to ^1^H
a	130.09	2	CH	A
b	61.18	1	CH_2_	C
c	33.03	1	CH_2_	F
d	31.76	1	CH_2_	H
e	29.58	1	CH_2_	H
f	29.56	2	CH_2_	G
g	29.50	1	CH_2_	H
h	29.45	1	CH_2_	H
i	29.35	1	CH_2_	H
j	29.31	1	CH_2_	H
k	29.17	1	CH_2_	H
l	29.06	2	CH_2_	H
m	27.03	2	CH_2_	E
n	25.99	1	CH_2_	H
o	22.57	1	CH_2_	H
p	14.40	1	CH_3_	I

## Supplementary Materials

The following are available online at www.mdpi.com/2076-393X/4/2/11/s1.
