# Supplementary material for: Effect of Bee Venom and Its Fractions on the Release of Pro-Inflammatory Cytokines in PMA-Differentiated U937 Cells Co-Stimulated with LPS
Source: Vaccines (Basel). 2016 Apr 19;4(2):11. doi: 10.3390/vaccines4020011 (PMC4931628; doi:10.3390/vaccines4020011)
Supplement: Supplementary file 1 [file vaccines-04-00011-s001.docx]

**Supplementary Materials:** **Effect of Bee Venom and Its Fractions on the Release of Pro-Inflammatory Cytokines in PMA-Differentiated U937 Cells
Co-Stimulated with LPS**

Jonans Tusiimire, Jennifer Wallace, Nicola Woods, Mark J. Dufton, John A. Parkinson,
Grainne Abbott, Carol J. Clements, Louise Young, Jin Kyu Park, Jong Woon Jeon,
Valerie A. Ferro and David G. Watson

Supplementary Experimental Details: NMR Spectroscopy

NMR spectroscopy studies were carried out on 7.4 mg of a sample of fraction F4 from BV. This was dissolved in 600 μL DMSO-*d*_6_ contained in a 5 mm Ø standard NMR tube.

NMR data were acquired on a Bruker AVANCE II^+^ NMR spectrometer equipped with a 14.1 T Ultrashield superconducting magnet operating at a ^1^H resonance frequency of 600.13 MHz. All data were acquired using a BBO-z-atm probehead operating at ambient temperature (298 K) regulated by means of a BCU-05 chiller unit.

1D ^1^H-NMR spectra were acquired with 16 transients over a frequency width of 7.2 kHz
(12.0 ppm) centred at a frequency offset of 5.0 ppm into 32 K data points for an acquisition time of 2.27 s using a 30 degree radio frequency (r.f.) pulse and a recycle delay of 2.0 s.

1D ^13^C-{^1^H} NMR spectra were acquired with 1024 transients over a frequency width of
33.33 kHz (220.8 ppm) centred at a frequency offset of 100.0 ppm into 32 K data points for an acquisition time of 491.5 ms using a 30 degree r.f. pulse with continuous composite pulse decoupling applied at the ^1^H resonance frequency and using a recycle delay of 0.7 s.

2D [^1^H, ^1^H] phase-sensitive double quantum filtered COSY and TOCSY NMR data were acquired with 8 and 4 transients respectively for each of 360 TPPI t_1_ increments into 2 K complex data points over ω_2_ and ω_1_ frequency widths of 3.6 kHz (6 ppm, ω_2_ acquisition time = 284.7 ms) centred at a frequency offset of 3.0 ppm and using zero-quantum suppression to reduce interference from zero-quantum effects.

2D [^1^H, ^13^C] HSQC-NMR spectra were acquired in both traditional mode and over a reduced
ω_1_ frequency width using non-uniform sampling (NUS) to increase F1 resolution. Traditional low resolution NMR data were acquired using a multiplicity edited, echo-antiecho gradient selected approach with sensitivity improvement (Bruker pulse program hsqcedetgpsisp2.3). Data were acquired into 2 K complex data points over an ω_2_ frequency width of 3.6 kHz (6.0 ppm) centred at a frequency offset of 3.0 ppm and an ω_1_ frequency width of 24.9 kHz (165.0 ppm) centred at a frequency offset of 70.0 ppm with 8 transients for each of 256 t_1_ increments. High resolution data were acquired with the ω_1_ frequency width set to 4.5 kHz (30.0 ppm) centred at a frequency offset of 30.0 ppm nominally using 2048 t_1_ increments sampled at 25% NUS (equivalent to 256 NUS t_1_ increments) using a random generator seed of 54321 for NUS purposes.

2D [^1^H, ^13^C] HMBC-NMR spectra were also acquired in both traditional and NUS modes into
2 K complex data points over an ω_2_ frequency width of 3.6 kHz (6.0 ppm) centred at a frequency offset of 3.0 ppm and an ω_1_ frequency width of 33.55 kHz (222.4 ppm) centred at a frequency offset of
100.0 ppm with 8 transients for each of 256 traditional and NUS (25% sampled) t_1_ increments respectively. Data were acquired with gradient coherence selection and used a low-pass filter to reduce the appearance of artefacts from ^1^*J*_HC_ coupling.

NUS data were processed using the MDD (multidimensional decomposition) algorithm developed by Orekhov *et al.* followed by Hilbert transformation to allow traditional phase correction and processing of the data.

**Figure S1.** A representative 4-parameter logistic plot of TNF-α standard samples showing the calibration equation and the values of the constants a, b, c, and d with a perfect fit R2 values of 1.000. Error bars represent standard deviation of absorbance values for duplicate standard concentrations (*n* = 2).

**Figure S2.** A representative 4-parameter logistic plot of TNF-α standard samples showing the calibration equation and the values of the constants a, b, c, and d with a perfect fit R2 values of 1.000. Error bars represent standard deviation of absorbance values for duplicate standard concentrations (*n* = 2).

**Figure S3.** A representative 4-parameter logistic plot of TNF-α standard samples showing the calibration equation and the values of the constants a, b, c, and d with a perfect fit with R^2^ values of 1.000. Error bars represent standard deviation of absorbance values of 1.000. Error bars represent standard deviation of absorbance values triplicate standard concentrations (*n* = 3).

**Figure S4.** A sample 4-parameter logistic plot of the IL-1β/IL-1F2 standard samples showing the calibration equation and the values of the constants a, b, c, and d with a perfect fit R2 values of 1.000. Error bars represent standard deviation of absorbance values for duplicate standard concentrations (*n* = 2).

**Figure S5.** A sample 4-parameter logistic plot of the IL-1β/IL-1F2 standard samples showing the calibration equation and the values of the constants a, b, c, and d with a perfect fit R2 values of 1.000. Error bars represent standard deviation of absorbance values for duplicate standard concentrations (*n* = 2).

**Figure S6.** A sample 4-parameter logistic plot of the IL-1β/IL-1F2 standard samples showing the calibration equation and the values of the constants a, b, c, and d with a perfect fit R^2^ values of 1.000. Error bars represent standard deviation of absorbance values for triplicate standard concentrations
(*n* = 3).

**Figure S7.** A sample 4-parameter logistic plot of the IL-6 standard samples showing the calibration equation and the values of the constants a, b, c, and d with a perfect fit R^2^ values of 1.000. Error bars represent standard deviation of absorbance values for duplicate standard concentrations (*n* = 2).

**Figure S8.** A sample 4-parameter logistic plot of the IL-6 standard samples showing the calibration equation and the values of the constants a, b, c, and d with a perfect fit R^2^ values of 1.000. Error bars represent standard deviation of absorbance values for duplicate standard concentrations (*n* = 2).

**Figure S9.** A sample 4-parameter logistic plot of the IL-6 standard samples showing the calibration equation and the values of the constants a, b, c, and d with a perfect fit R^2^ values of 1.000. Error bars represent standard deviation of absorbance values for duplicate standard concentrations (*n* = 2).

**Table S1.** Volumes of LPS, fractions and media used to stimulate cytokine production in
PMA-differentiated U937 cells.

| **Sample** | **Volume Added (µL)** | | | **Concentration (μg/mL)** | |
| --- | --- | --- | --- | --- | --- |
|  | ***Stock Solution (10 mg/mL)*** | ***LPS (1 mg/mL)*** | ***Media*** | ***Fraction*** | ***LPS*** |
| F1 | 80.0 | 8.0 | 1912.0 | 400.0 | 4.0 |
| F2 | 80.0 | 8.0 | 1912.0 | 400.0 | 4.0 |
| F3 | 2.4 | 8.0 | 1989.6 | 12.0 | 4.0 |
| F4 | 24.0 | 8.0 | 1968.0 | 120.0 | 4.0 |
| BV | 2.4 | 8.0 | 1989.6 | 12.0 | 4.0 |
| LPS | - | 8.0 | 1992.0 | - | 4.0 |

**Table S2.** The constant values of the 4-PL regression curve fitted to the data of TNF-α, IL-1β and
IL-6 standards respectively (*n* = 3).

| **Constants** | **TNF-α** | | | **IL-1β** | | | **IL-6** | | |
| --- | --- | --- | --- | --- | --- | --- | --- | --- | --- |
| *Assay* | *n = 1* | *n = 2* | *n = 3* | *n = 1* | *n = 2* | *n = 3* | *n = 1* | *n = 2* | *n = 3* |
| a | 0.265 | 0.194 | 0.114 | 0.132 | 0.184 | 0.103 | 0.104 | 0.0708 | 0.0377 |
| b | 1.11 | 1.07 | 1.2 | 1.02 | 1.14 | 1.25 | 1.33 | 1.08 | 1.16 |
| c | 711 | 1180 | 541 | 191 | 222 | 136 | 165 | 444 | 364 |
| d | 5.15 | 6.03 | 4.6 | 5.72 | 5.24 | 4.35 | 3.95 | 5.21 | 4.82 |
| R^2^ | 1.000 | 1.000 | 1.000 | 1.00 | 1.000 | 1.000 | 0.999 | 1.000 | 1.000 |

**Table S3.** Concentrations of the fractions used in the assay of immuno-modulatory effects on
PMA-differentiated U937 cells. For each fraction, the concentration selected was the highest
non-toxic one.

| **SN** | **Fraction** | **IC50 (μg/mL)** | **Selected Concentration (μg/mL)** |
| --- | --- | --- | --- |
| 1 | F-1 | >100 | 100 |
| 2 | F-2 | >100 | 100 |
| 4 | F-3 | 5.4 | 3.0 |
| 3 | F-4 | 68.8 | 30.0 |

**Table S4.** Effect of bee venom and its fractions on TNF-α production in differentiated U937 cells in the presence or absence of LPS co-stimulation (*n* = 3).

| **Samples** | **TNF-α Concentration (pg/mL)** | | | | | | | | | |
| --- | --- | --- | --- | --- | --- | --- | --- | --- | --- | --- |
| **Samples** | **Sample−LPS** | | | | | **Sample+LPS** | | | | |
|  | ***n* = 1** | ***n* = 2** | ***n* = 3** | **Mean** | **SD** | ***n* = 1** | ***n* = 2** | ***n* = 3** | **Mean** | **SD** |
| F-1 | 6.1 | 6.8 | 3.8 | 5.6 | 1.6 | 68.2 | 126.8 | 70.9 | 88.6 | 33.1 |
| F-2 | 5.7 | 7.2 | 4.4 | 5.8 | 1.4 | 74.1 | 162.0 | 90.8 | 109.0 | 46.7 |
| F-3 | 7.6 | 8.9 | 5.2 | 7.2 | 1.9 | 69.5 | 161.5 | 84.2 | 105.1 | 49.4 |
| F-4 | 8.0 | 9.7 | 8.0 | 8.6 | 1.0 | 88.1 | 160.1 | 143.1 | 130.4 | 37.6 |
| BV | 8.2 | 7.8 | 5.0 | 7.0 | 1.7 | 56.0 | 79.0 | 81.0 | 72.0 | 13.9 |

**TNF-α Controls**

| **Control** | **TNF-α Concentration (pg/mL)** | | | | |
| --- | --- | --- | --- | --- | --- |
| *Replicate No.* | *n = 1* | *n = 2* | *n = 3* | *Mean* | *SD* |
| Media | 7.3 | 7.1 | 4.4 | 6.2 | 1.6 |
| LPS | 66.2 | 114.9 | 73.0 | 84.7 | 26.4 |

**Table S5.** Effect of bee venom and its fractions on IL-1β/IL-1F2 production in differentiated U937 cells in the presence or absence of LPS co-stimulation (*n* = 3).

| **Samples** | **IL-1β (pg/mL)** | | | | | | | | | |
| --- | --- | --- | --- | --- | --- | --- | --- | --- | --- | --- |
|  | ***Sample−LPS*** | | | | | ***Sample+LPS*** | | | | |
|  | ***n* = 1** | ***n* = 2** | ***n* = 3** | **Mean** | **SD** | ***n* = 1** | ***n* = 2** | ***n* = 3** | **Mean** | **SD** |
| F-1 | 1.2 | 1.7 | 0.5 | 1.1 | 0.6 | 34.4 | 293.1 | 86.9 | 138.1 | 136.7 |
| F-2 | 1.2 | 2.2 | 0.8 | 1.4 | 0.7 | 53.4 | 703.1 | 174.3 | 310.2 | 345.6 |
| F-3 | 1.1 | 1.8 | 1.3 | 1.4 | 0.4 | 78.3 | 279.8 | 108.3 | 155.5 | 108.7 |
| F-4 | 0.8 | 3.6 | 3.4 | 2.6 | 1.5 | 27.1 | 137.7 | 71.7 | 78.8 | 55.6 |
| BV | <2.0 | 2.8 | 2.4 | 2.6 | 0.3 | 24.7 | 83.3 | 45.9 | 51.3 | 29.7 |

**IL-1β Controls**

| **Control** | **IL-1β Concentration (pg/mL)** | | | | |
| --- | --- | --- | --- | --- | --- |
| *Replicate No.* | *n = 1* | *n = 2* | *n = 3* | *Mean* | *SD* |
| Media | <2.0 | 1.2 | 2.5 | 1.8 | 0.9 |
| LPS | 9.1 | 49.1 | 22.0 | 26.8 | 20.4 |

**Table S6.** Effect of bee venom and its fractions on IL-6 production in differentiated U937 cells in the presence and absence of LPS co-stimulation (*n* = 3).

| **Samples** | **IL-6 Concentration (pg/mL)** | | | | | | | | | |
| --- | --- | --- | --- | --- | --- | --- | --- | --- | --- | --- |
|  | ***Sample−LPS*** | | | | | ***Sample+LPS*** | | | | |
|  | ***n* = 1** | ***n* = 2** | ***n* = 3** | **Mean** | **SD** | ***n* = 1** | ***n* = 2** | ***n* = 3** | **Mean** | **SD** |
| F-1 | <4.7 | <4.7 | <4.7 | <4.7 | n/a | 93.8 | 199.1 | 126.6 | 139.8 | 53.9 |
| F-2 | <4.7 | <4.7 | <4.7 | <4.7 | n/a | 113.1 | 235.6 | 160.6 | 169.8 | 61.8 |
| F-3 | <4.7 | <4.7 | <4.7 | <4.7 | n/a | 112.1 | 159.0 | 152.1 | 141.1 | 25.3 |
| F-4 | <4.7 | <4.7 | <4.7 | <4.7 | n/a | 32.3 | 63.7 | 71.4 | 55.8 | 20.7 |
| BV | <4.7 | <4.7 | <4.7 | <4.7 | n/a | 98.5 | 198.6 | 183.9 | 160.3 | 54.0 |

**IL-6 Controls**

| **Control** | **IL-6 Concentration (pg/mL)** | | | | |
| --- | --- | --- | --- | --- | --- |
| *Replicate No.* | *n = 1* | *n = 2* | *n = 3* | *Mean* | *SD* |
| Media | <4.7 | <4.7 | <4.7 | <4.7 | n/a |
| LPS | 58.5 | 185.5 | 145.8 | 129.9 | 65.0 |

Additional LC-MS Data





**Figure S10.** Extracted ion chromatograms (EICs) of the main components of F-1. The fraction was analysed by positive ion LC-ESI-MS on the Orbitrap mass spectrometer with an ACE 3 C18 column using mobile phases 0.1% formic acid in water (A) and 0.1% formic acid in acetonitrile (B) at a gradient of 20%–70% (0–10 min), 70% (10–16 min), 70%–20% (16–20 min) and 20% (20–25 min) relative to solvent B, and at a flow rate of 0.3 mL/min. The EICs were obtained within ±0.01 accuracy relative to the *m*/*z* of each ion.





**Figure S11.** Full scan (**a**) and extracted ion chromatograms (**b**–**e**) of the components of F-2. The fraction was analysed by positive ion LC-ESI-MS on the Orbitrap mass spectrometer with an ACE 3 C18 column using mobile phases 0.1% formic acid in water (A) and 0.1% formic acid in acetonitrile (B) at a gradient of 20%–70% (0–10 min), 70% (10–16 min), 70%–20% (16–20 min) and 20% (20–25 min) relative to solvent B, and at a flow rate of 0.3 mL/min.





**Figure S12.** Chromatogram of sample from F-3 showing the melittin peak in a relatively pure form (A). The typical mass spectrum of melittin (B) shows a quintuply-charged base ion with *m*/*z* 570.1585. The fraction was analysed by positive ion LC-ESI-MS on the Orbitrap mass spectrometer with an ACE 3 C18 column using mobile phases 0.1% formic acid in water (A) and 0.1% formic acid in acetonitrile (B) at a gradient of 20%–70% (0–10 min), 70% (10–16 min), 70%–20% (16–20 min) and 20% (20–25 min) relative to solvent B, and at a flow rate of 0.3 mL/min.





**Figure S13.** Chromatogram of the impurity in F-4 with [M − H] of 793.4651 and elemental composition C_43_H_70_O_11_P. Collisional-induced dissociation (CID) of *m*/*z* 793.46 parental ion at a NCE of 35.0 gave two fragment ions of *m*/*z* 493.2574 and 643.3608 respectively. Sample analysed by LCMS using mobile phase A (0.1% formic acid in water), B (0.1% formic acid in acetonitrile), gradient [50%–95% B
(0–5 min), 95% B (5–12 min), 50% B (13–16 min)], flow rate 0.4 mL/min, column ACE 3 C18 (150 × 3.0 mm), 3 µm i.d. The ions were detected on the LTQ-Orbitrap (*m*/*z* scan range 200–1200 for parental ion and 200–700 for fragment ions) in negative mode.





**Figure S14.** 600 MHz 1D ^1^H-NMR spectrum of the lipid component from faction F-4 showing signal integrals. The signal indicated by * arises from the solvent and signal D is ascribed to water in DMSO-*d*_6_.





**Figure S15.** ^13^C-{^1^H} NMR spectrum of the lipid component from fraction F-4 with expansions shown to reveal details of crowded region. The expansion immediately above the full NMR spectrum shows expansion of the signal region c–p. Further expansion of the region e–l within this is shown inset as the topmost expansion.


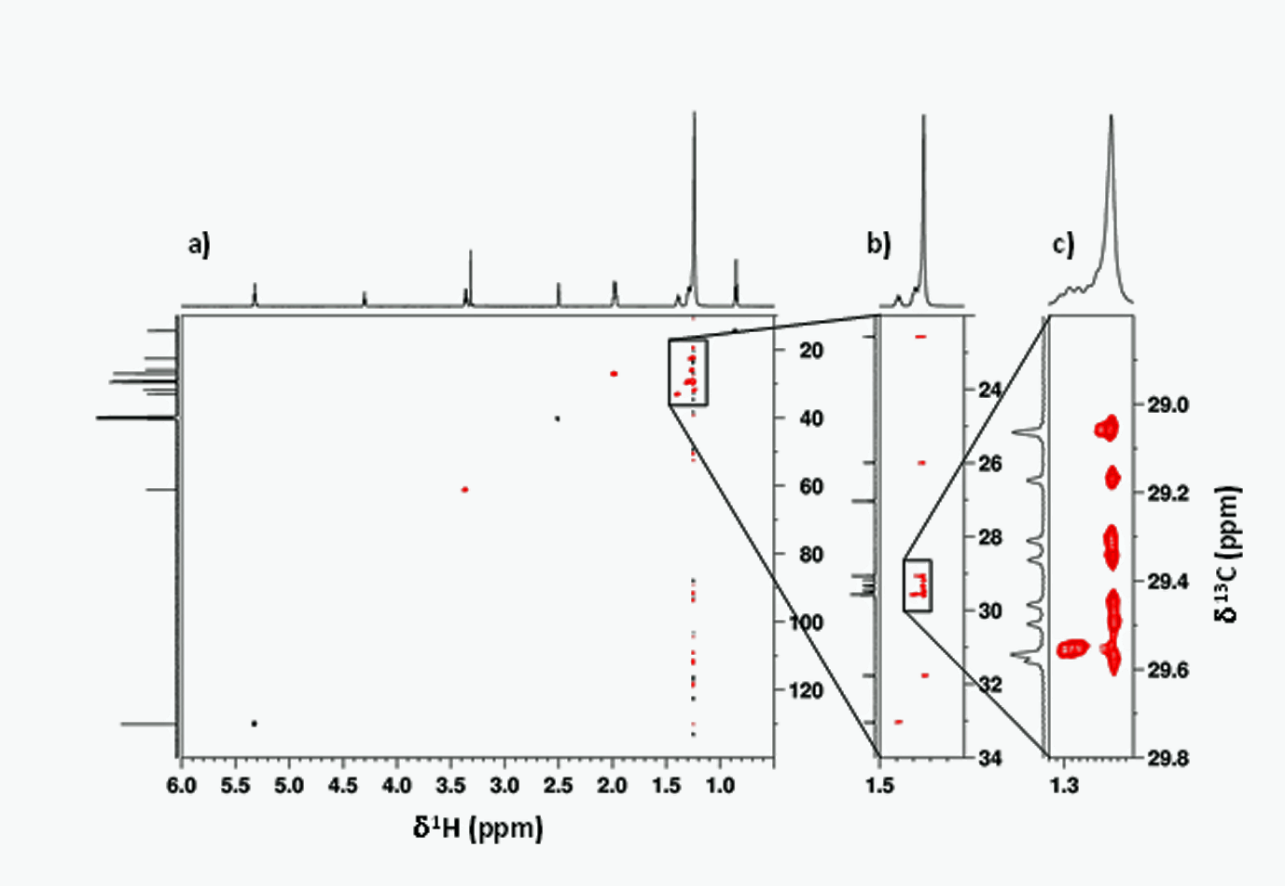


**Figure S16.** 600 MHz 2D [^1^H, ^13^C] HSQC-NMR data for the lipid component of fraction F-4. (**a**) Low resolution spectrum showing the complete data set; (**b**) Expansion of the boxed region shown in (**a**) from data acquired at high F1 resolution using non-uniform sampling; (**c**) Further expansion of the boxed region shown in (**b**) to reveal high resolution data in F1 allowing individual correlations between protons and carbons of each methylene group to be distinguished for unique carbon resonance assignment and proton counting. Black cross-peaks (positive) correspond to methine (CH) and methyl (CH_3_) groups; red cross-peaks correspond to methylene (CH_2_) groups.

**Figure S17.** 600 MHz 2D [^1^H, ^1^H] DQFCOSY NMR spectrum of the BV F4 component molecule with 1D ^1^H-NMR spectra shown representing horizontal and vertical projections with signal designations shown as **A**–**I**. Selected cross-peaks are highlighted. Full cross-peak assignments are summarized in Table S7.

**Figure S18.** 600 MHz 2D [^1^H, ^1^H] TOCSY NMR spectrum of the BV F4 component molecule with 1D ^1^H-NMR spectra shown representing horizontal and vertical projections with signal designations shown as **A**–**I**. Full cross-peak assignments are summarized in Table S7.

**Figure S19.** 600 MHz 2D [^1^H, ^13^C] HMBC-NMR spectrum of the BV F4 component molecule with 1D ^1^H-NMR spectrum shown representing the horizontal projection and 1D ^13^C-{^1^H} NMR spectrum shown representing the vertical projection with signal designations shown as **A**–**I** and **a**–**p** respectively. Full cross-peak assignments are summarized in Table S8.





**Figure S20.** Basic lipid structure from an analysis of the NMR data showing the signal assignments associated with protons (upper case labels) and carbons (lower case labels) at each position. The
*E*-layout about the double bond is purely for convenience and has no bearing on the final structure.





**Figure S21.** Simulations and experimental data for two possible configurations of the lipid molecule for ^1^H resonances A (**left**) and E (**right**). (**a**) Simulated signals for the *E*-configuration; (**b**) simulated signals for the *Z*-configuration; (**c**) experimental data.

**Table S7.** Coupling partners revealed through 2D [^1^H, ^1^H] COSY (C) and TOCSY (T) NMR data

| **Label** | **A** | **B** | **C** | **D** | **E** | **F** | **G** | **H** | **I** |
| --- | --- | --- | --- | --- | --- | --- | --- | --- | --- |
| **A** |  |  |  |  | C, T |  |  |  |  |
| **B** |  |  | C, T |  |  |  |  |  |  |
| **C** |  | C, T |  |  |  | C, T |  |  |  |
| **D** |  |  |  |  |  |  |  |  |  |
| **E** | C, T |  |  |  |  |  | C, T | C, T |  |
| **F** |  |  | C, T |  |  |  |  | C, T |  |
| **G** |  |  |  |  | C, T |  |  | C, T |  |
| **H** |  |  |  |  | C, T | C, T | C, T |  | C, T |
| **I** |  |  |  |  |  |  |  | C, T |  |

**Table S8.** Coupling partners revealed by analysis of 2D [^1^H, ^13^C] HSQC (HS) and HMBC (HM) NMR data for the lipid component.

| **Labels** | **A** | **B** | **C** | **D** | **E** | **F** | **G** | **H** | **I** |
| --- | --- | --- | --- | --- | --- | --- | --- | --- | --- |
| **a** | HS |  |  |  | HM |  | HM |  |  |
| **b** |  | HM | HS |  |  | HM |  | HM |  |
| **c** |  | HM | HM |  |  | HS |  | HM |  |
| **d** |  |  |  |  |  |  |  | HSHM | HM |
| **e** |  |  |  |  |  |  |  | HS |  |
| **f** | HM |  |  |  | HM |  | HS |  |  |
| **g** |  |  |  |  |  |  |  | HS |  |
| **h** |  |  |  |  |  | HM |  | HS |  |
| **i** |  |  |  |  |  |  |  | HS |  |
| **j** |  |  |  |  |  |  |  | HS |  |
| **k** |  |  |  |  |  |  |  | HS |  |
| **l** |  |  |  |  | HM |  | HM | HS |  |
| **m** | HM |  |  |  | HS |  | HM | HM |  |
| **n** |  |  | HM |  |  | HM |  | HS |  |
| **o** |  |  |  |  |  |  |  | HS | HM |
| **p** |  |  |  |  |  |  |  | HM | HS |





**Figure S22.** (*Z)*-eicos-9-en-1-ol.

References

1. Thrippleton, M.J.; Keeler, J. Elimination of Zero-Quantum Interference in Two-Dimensional NMR Spectra. *Angew. Chem. Int. Ed.* **2003**, *42*, 3938–3941
2. Jaravine, V.; Zhuravleva, A.; Permi, P.; Ibraghimov, I.; Orekhov, V.Y. Hyperdimensional NMR spectroscopy with nonlinear sampling. *J. Am. Chem. Soc.* **2008**, *130*, 3927–3936.

© 2016 by the authors; licensee MDPI, Basel, Switzerland. This article is an open access article distributed under the terms and conditions of the Creative Commons by Attribution (CC-BY) license (http://creativecommons.org/licenses/by/4.0/).
